# ID 142 - Uso do DataSUS na Avaliação da Implementação do PCDT de Transtorno Bipolar

**DOI:** 10.5327/2237-9622.2025.v34s1.142

**Published:** 2025-11-25

**Authors:** Ísis Nalin Fernandes Nonato, Ana Laura de Sene Amâncio Zara, Adriane Lopes Medeiros Simone, Daniela Oliveira de Melo

**Keywords:** protocolos clínicos, guia de prática clínica, sistemas de informação em saúde, transtorno afetivo bipolar

## Abstract

**Introdução:**

No Brasil, o Sistema Único de Saúde (SUS) enfrenta uma série de desafios complexos que afetam a qualidade e o alcance dos serviços de saúde. Consequentemente, discrepâncias na implementação de procedimentos recomendados em Protocolos Clínicos e Diretrizes Terapêuticas (PCDT) prejudicam o tratamento de diversas doenças, como o transtorno afetivo bipolar (TAB). Este estudo teve como objetivo analisar, a partir de dados secundários, alterações no padrão de consumo de medicamentos do PCDT de TAB no período de março de 2016 a dezembro de 2023 no Brasil.

**Método:**

Os dados sobre a dispensação ambulatorial de antipsicóticos atípicos (quetiapina, olanzapina, risperidona, clozapina) e lamotrigina foram baixados do Sistema de Informações Ambulatoriais do SUS (SIA-SUS), disponíveis no site do Departamento de Informática do Sistema Único de Saúde (DataSUS). As variáveis de interesse foram extraídas utilizando o programa TabWin e manipuladas para realização das análises descritivas. Indicadores de dose diária definida (DDD) foram calculados e uma análise de tendência do número de dispensações foi realizada usando a regressão Prais-Winsten (p<0,05).

**Resultados:**

Entre março/2016 e dezembro/2023, foram identificadas 4.290.165 dispensações de medicamentos para o tratamento de TAB no Brasil, especialmente nas Regiões Sudeste (43,67%) e Sul (30,42%). Todos os medicamentos apresentaram uma tendência crescente no número de dispensações e um aumento da DDD/100 mil habitantes/dia, especialmente em relação à quetiapina 200 mg (TIMM: 10,72%; IC 95%: 3,25-18,73; p=0,005) e olanzapina 5 mg (TIMM: 9,80%; IC 95%: 2,98-17,06; p=0,005). Entre 2016-2022, a DDD/100 mil habitantes/dia de quetiapina e olanzapina variou de 0,28 a 12,08 e 0,18 a 7,23, respectivamente. Em relação à idade, o número de dispensações de medicamentos para o tratamento de TAB em pacientes <18 anos foi crescente (974-37.715; p<0,05). Também foram identificadas dispensações de olanzapina e clozapina para pacientes pediátricos.

**Conclusão:**

A utilização de dados do SIA-SUS pode ser uma ferramenta valiosa na avaliação da implementação de PCDT no Brasil. A análise das séries temporais demonstrou a crescente tendência no consumo de antipsicóticos atípicos e lamotrigina para TAB, podendo refletir um aumento no acesso a medicamentos, mas também o aumento de diagnósticos de TAB, especialmente entre crianças e adolescentes. As dispensações de olanzapina e clozapina, medicamentos contraindicados para pacientes pediátricos, observadas em pacientes <18 anos, representam um alerta sobre como a idade dos pacientes está sendo considerada e controlada na dispensação de medicamentos pelas Farmácias Estaduais, uma vez que as restrições descritas no PCDT não foram refletidas na Tabela de Procedimentos, Medicamentos e OPM do SUS (Sigtap). Também foi observada uma grande discrepância no acesso da população a tratamentos entre as Regiões Sudeste e Sul, que concentraram 74,1% das dispensações, com as demais regiões, especialmente a Região Norte. Esse contexto sublinha a importância da adaptação de políticas de saúde pública no suprimento de particularidades regionais. O sucesso da implementação de PCDT no Brasil depende não apenas da disponibilidade de medicamentos, mas também da superação de desafios logísticos locais e da construção de uma rede de serviços que garanta o acesso equitativo a todos os brasileiros, independentemente de sua localização geográfica.

